# Supplementary material for: A widely conserved protein Rof inhibits transcription termination factor Rho and promotes Salmonella virulence program
Source: Nat Commun. 2024 Apr 15;15:3187. doi: 10.1038/s41467-024-47438-7 (PMC11018607; doi:10.1038/s41467-024-47438-7)
Supplement: Supplementary file 1 — Supplementary Information [file 41467_2024_47438_MOESM1_ESM.pdf]

Supplementary Information:

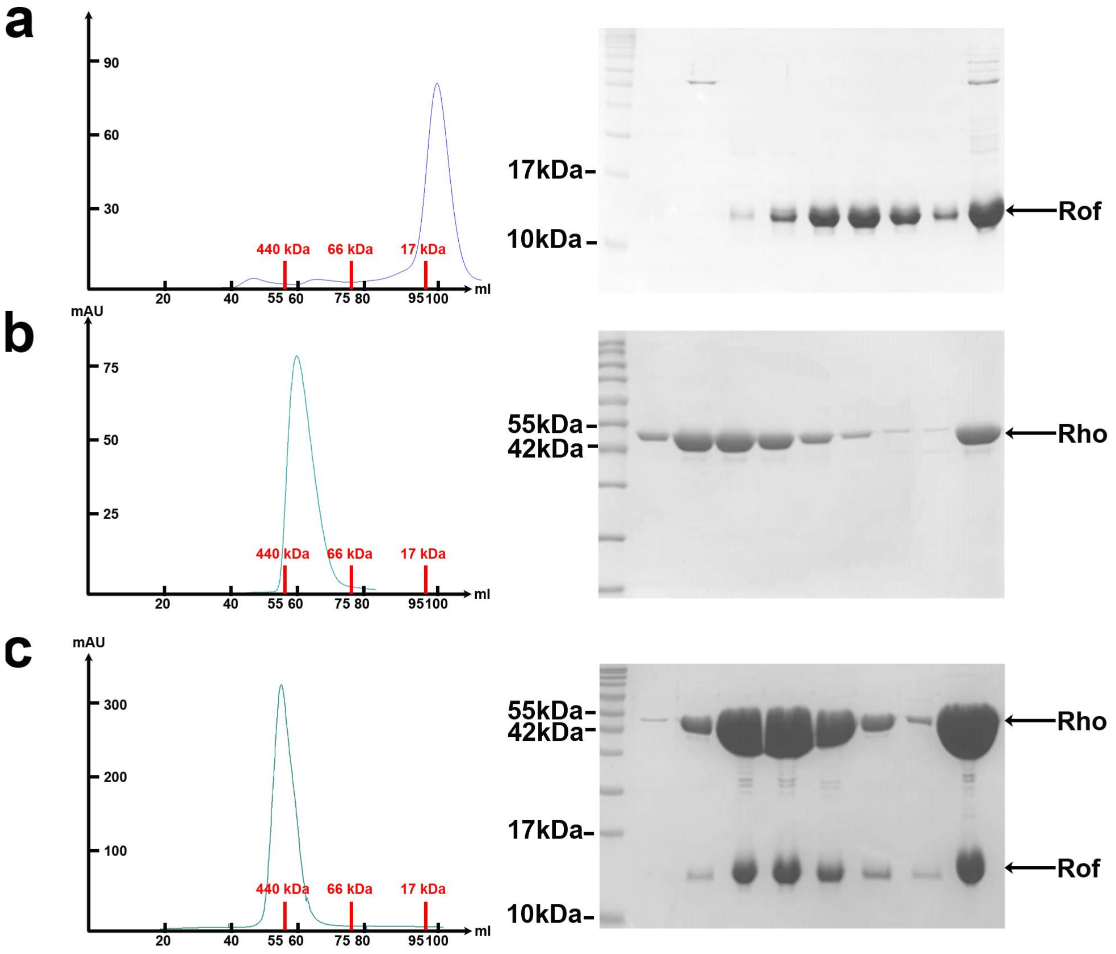

Supplementary Fig. 1. Purification and verification of *E. coli* Rof and Rho

(A) Left panel, Chromatogram map of gel filtration of *E. coli* Rof; right panel, SDS-PAGE of the purified *E. coli* Rof. Molecular weight standards are indicated as red vertical lines represent 440kDa, 66kDa, and 17kDa, respectively.

(B) Left panel, Chromatogram map of gel filtration of *E. coli* Rho; right panel, SDS-PAGE of the purified *E. coli* Rho. Molecular weight standards are indicated as red vertical lines represent 440kDa, 66kDa, and 17kDa, respectively.

(C) Left panel, Chromatogram map of gel filtration of *E. coli* Rho-Rof complex; right panel, SDS-PAGE of the purified *E. coli* Rho-Rof complex. Molecular weight standards are indicated as red vertical lines represent 440kDa, 66kDa, and 17kDa, respectively.

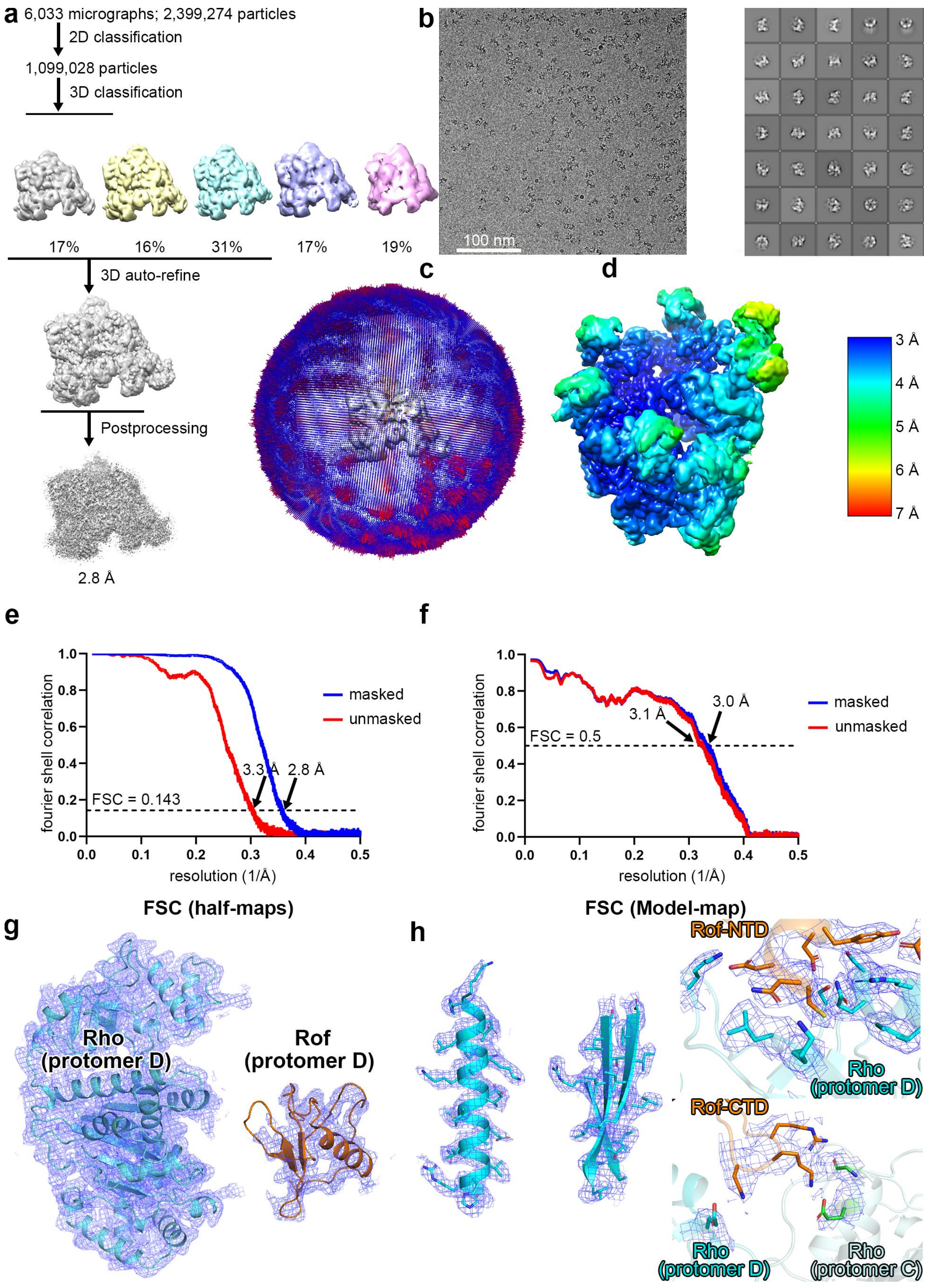

Supplementary Fig. 2. Structure determination of Rho-Rof antitermination complex

(A) Data processing scheme (Table S1).

(B) Representative electron micrograph and 2D class averages (50 nm scale bar in right subpanel).

(C) Orientation distribution.

(D) EM density map coloured by local resolution (horizontal inversion view as in Fig.2a, left).

(E) Fourier-shell-correlation (FSC = 0.143) plot for cryo-EM map.

(F) Fourier-shell-correlation (FSC = 0.5) plot for model-to-map.

(G) Representative EM density (blue mesh) and fits (ribbons) for Rho and Rof.

(H) Local density maps with different regions. Side chains are shown in sticks and structures are shown in cartoon.

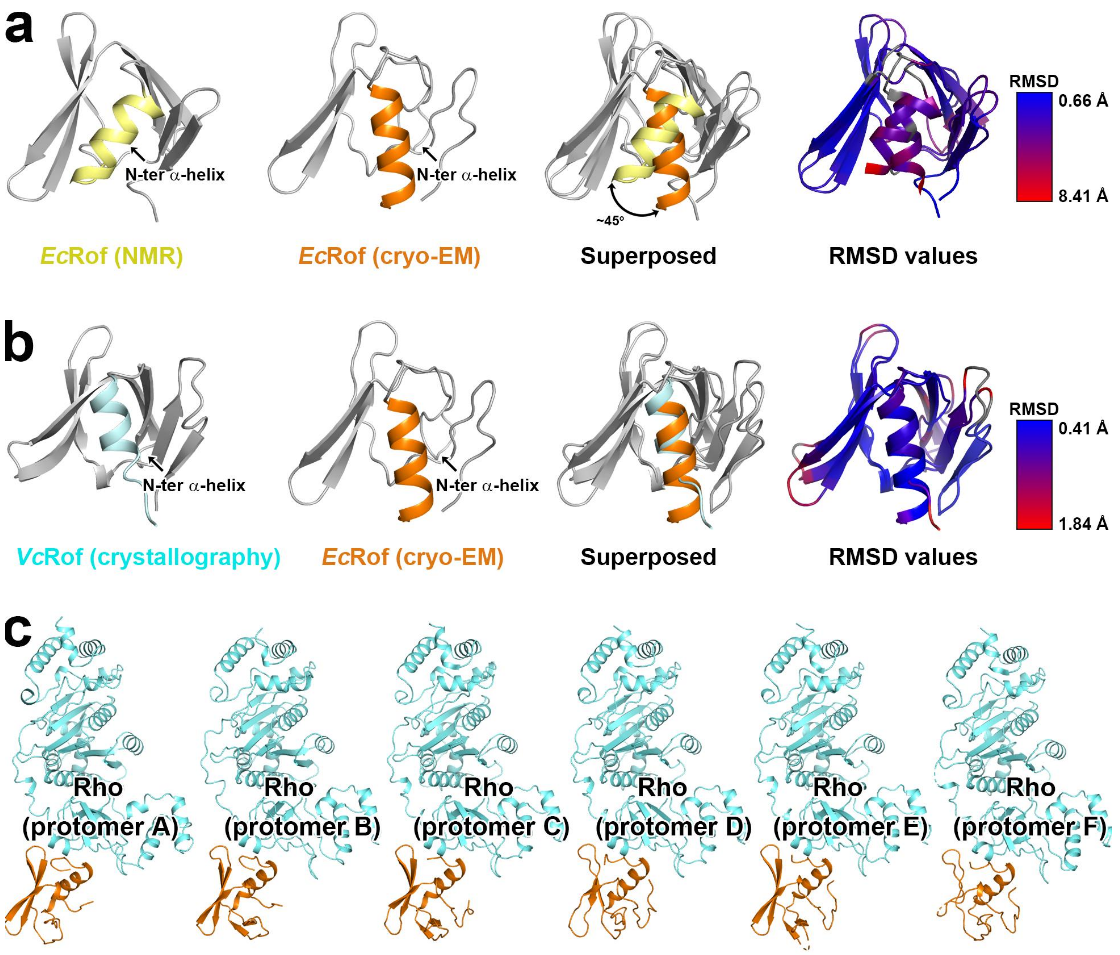

Supplementary Fig. 3. Structural comparison of Rof

(A) Superposition of *Ec*Rof (cryo-EM *vs* NMR). First panel, *Ec*Rof (Monomer, determined by NMR method, PDB ID 1SG5); second panel, *Ec*Rof (this work); third panel, Superposed models of *Ec*Rof (NMR) and *Ec*Rof (cryo-EM); fourth panel, Superposed models of *Ec*Rof (NMR) and *Ec*Rof (cryo-EM) colored by local RMSD value.

(B) Superposition of *Ec*Rof *vs* *Vc*Rof (PDB ID 6JIE). First panel, *Vc*Rof; second panel, *Ec*Rof (this work); third panel, Superposed models of *Vc*Rof (crystallography) and *Ec*Rof (cryo-EM); fourth panel, Superposed models of*Vc*Rof (crystallography) and *Ec*Rof (cryo-EM) colored by local RMSD value. *Vc* is short for *Vibrio cholerae*.

(C) Structures of Rof-Rho in each protomers A-F.

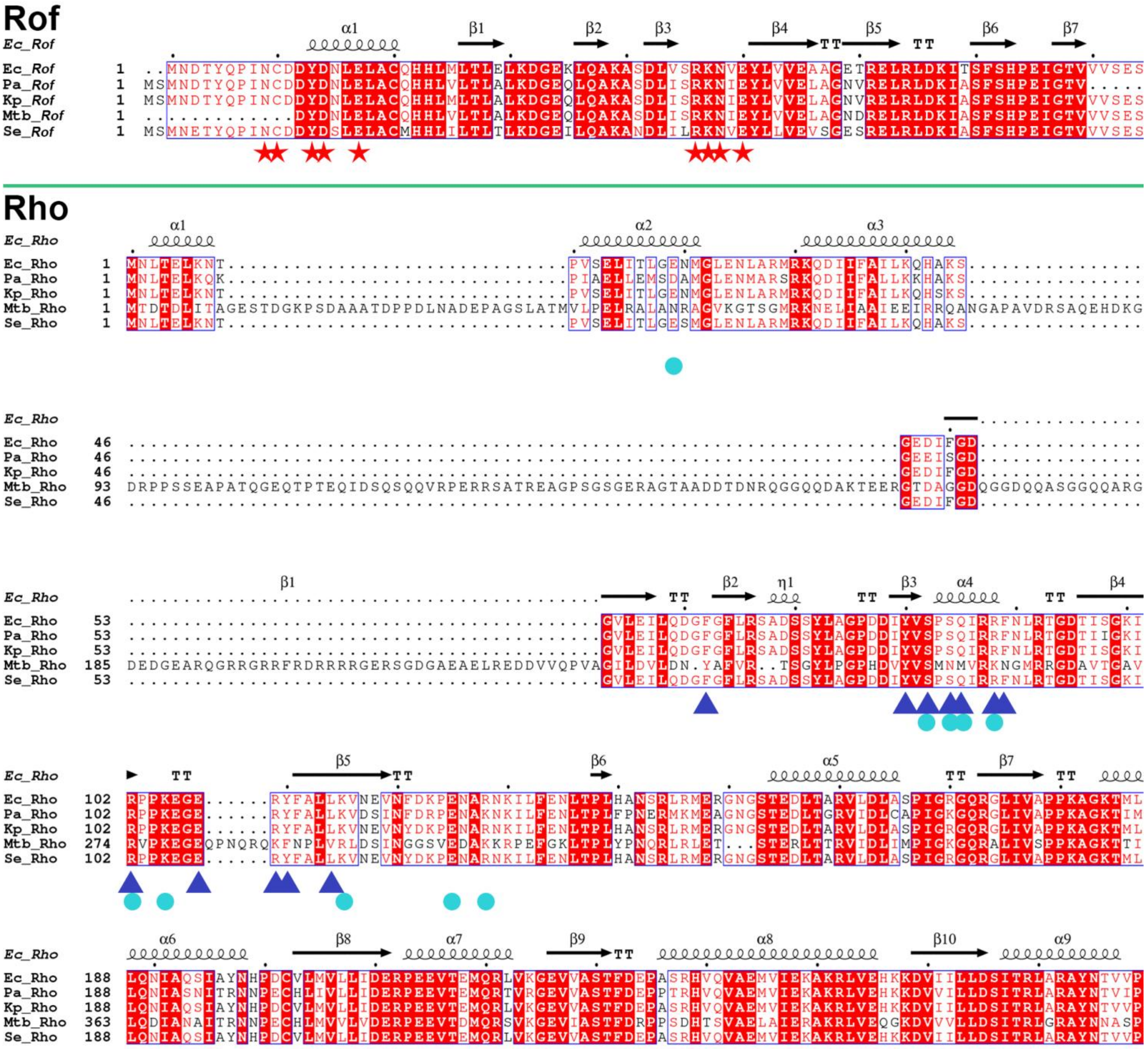

Supplementary Fig. 4. Sequence alignment of Rof and Rho.

Upper panel, amino acid sequence alignment of Rof from *Ec, Escherichia_coli; Pa, Pseudomonas aeruginosa; Kp, Klebsiella pneumoniae; Se, Salmonella enterica; Mtb, Mycobacterium tuberculosis*. The residues of Rof-Rho interface from Rof are indicated by red stars. Lower panel, amino acid sequence alignment of Rof from *Ec, Escherichia_coli; Pa, Pseudomonas aeruginosa; Kp, Klebsiella pneumoniae; Se, Salmonella enterica; Mtb, Mycobacterium tuberculosis*. The residues of Rof-Rho interface from Rho are marked by cyan circles and residues of Rho-RNA interface from Rho are indicated by blue triangles.

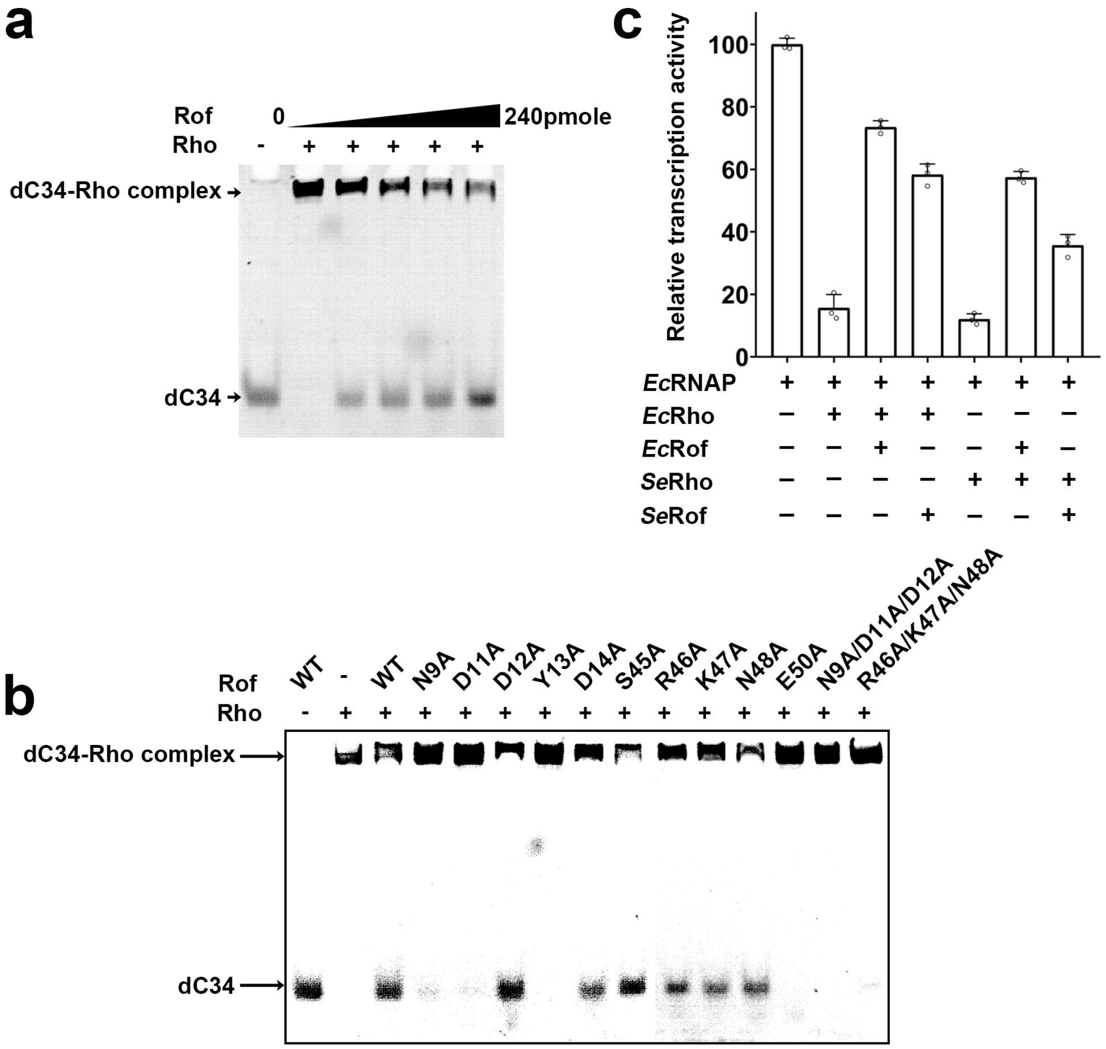

Supplementary Fig. 5. Electrophoretic mobility shift assay for Rho and PBS ligand RNA.

(A) EMSA assay for Rho and dC34 binding with or without Rof. Rho and 3μm dC34 were added into the reaction system. Bands of dC34, dC34-Rho complex are indicated by arrows on the left, respectively.

(B) EMSA assay for Rho and dC34 binding with or without Rof and its mutations. 3 uM Rho and 3 dC34 were added into the reaction system. Bands of dC34, dC34-Rho complex are indicated by arrows on the left, respectively.

(C) Relative in *vitro* transcription activity showing *Ec*Rof/*Ec*Rho/*Se*Rof/*Se*Rho are functional in Rho-dependent termination. Data for *in vitro* transcription assays are means of three technical replicates. Error bars represent ± SEM of n = 3 experiments. *Se, Salmonella enterica; Ec, Escherichia_coli.*

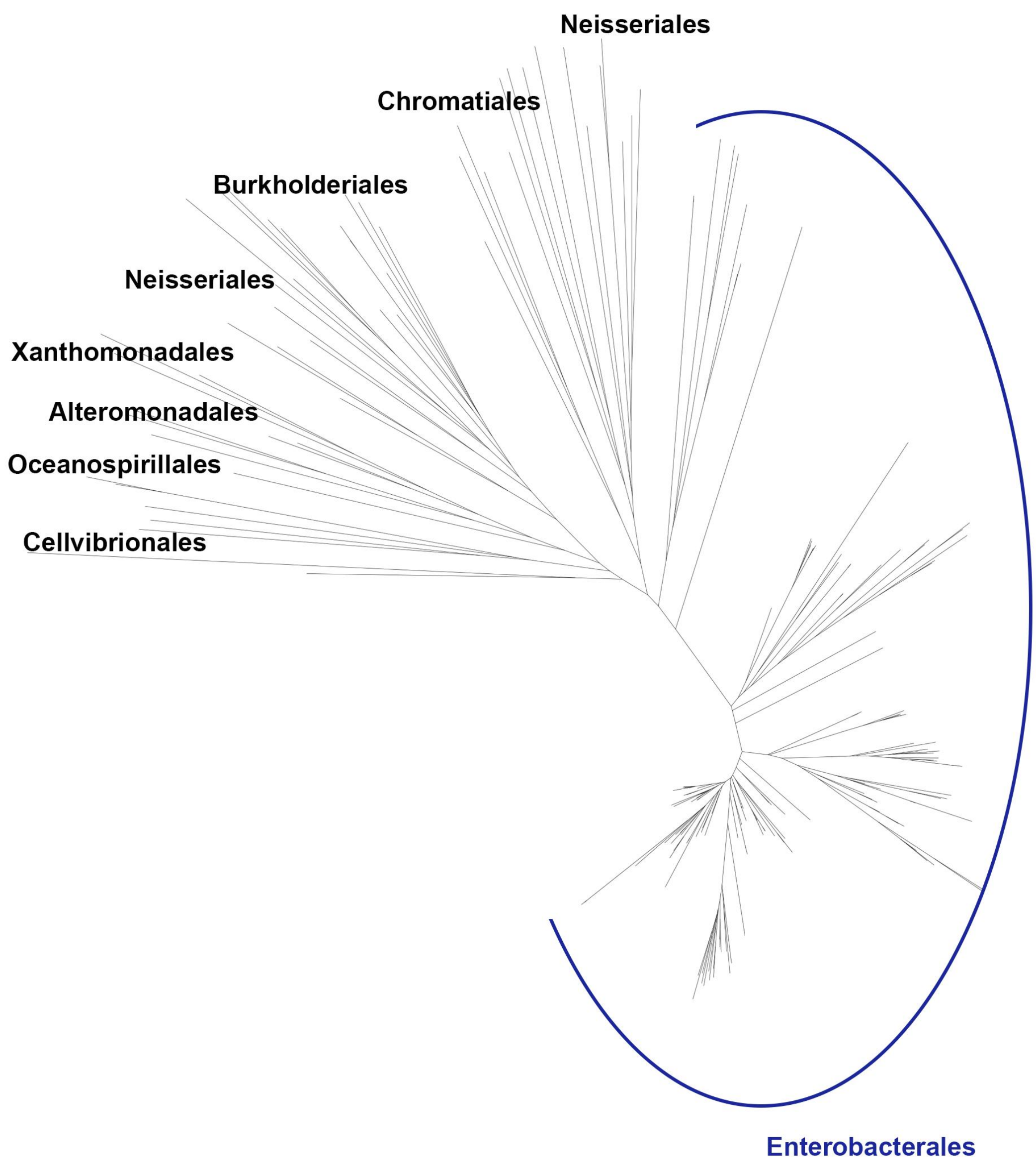

Supplementary Fig. 6. Phylogenetic analysis of the distribution of Rof in bacteria.

206 protein sequences of Rof are used in constructing the neighbor-joining phylogenetic tree. The family names of bacteria are shown on the top branch of the tree. The sequences belong to the Enterobacterales are highlighted with blue label and solid line.

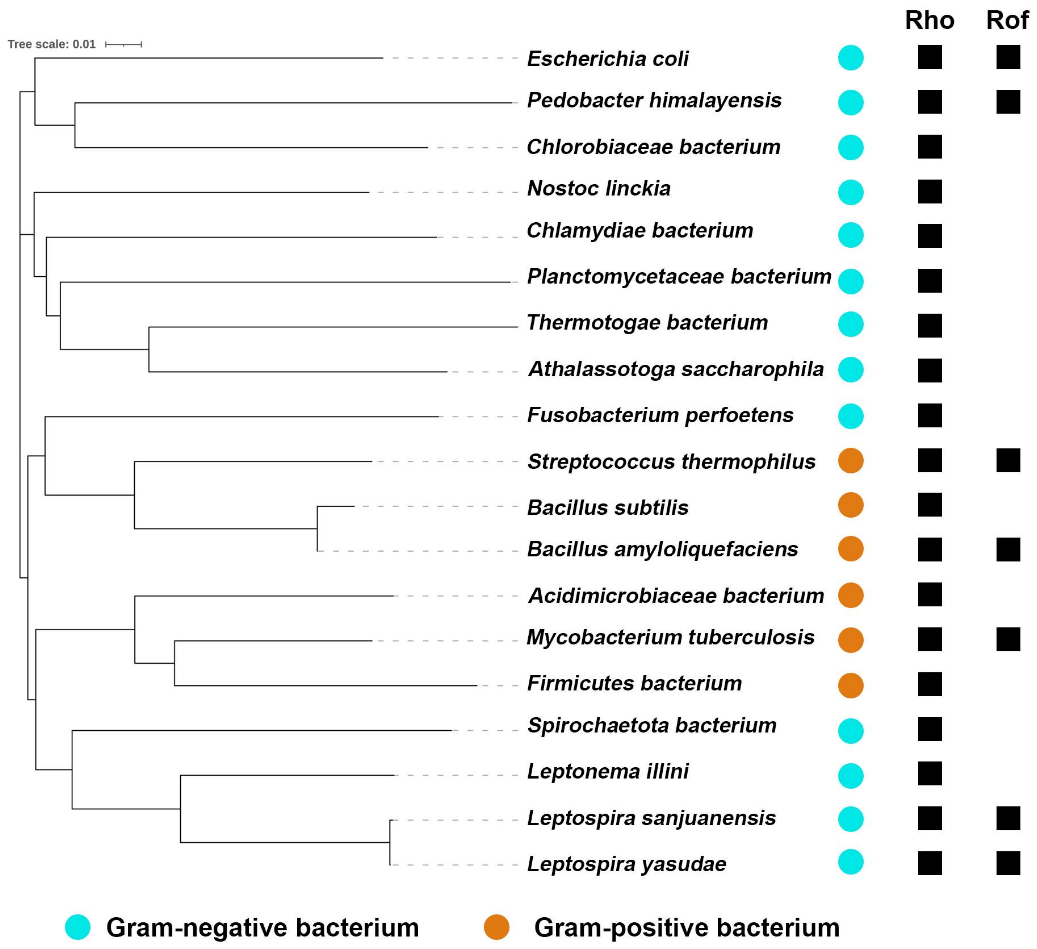

Supplementary Fig. 7. Phylogenetic analysis of the distribution of Rho and Rof in bacteria. 16s rRNA sequences from different species from Terrabacteria to α-proteobacteria are used in constructing the neighbor-joining phylogenetic tree. Gram-negative bacterium species are marked as cyan circles, and Gram-positive bacterium species are marked as orange circles. The genomes contains Rho or Rof are indicated as black squares.

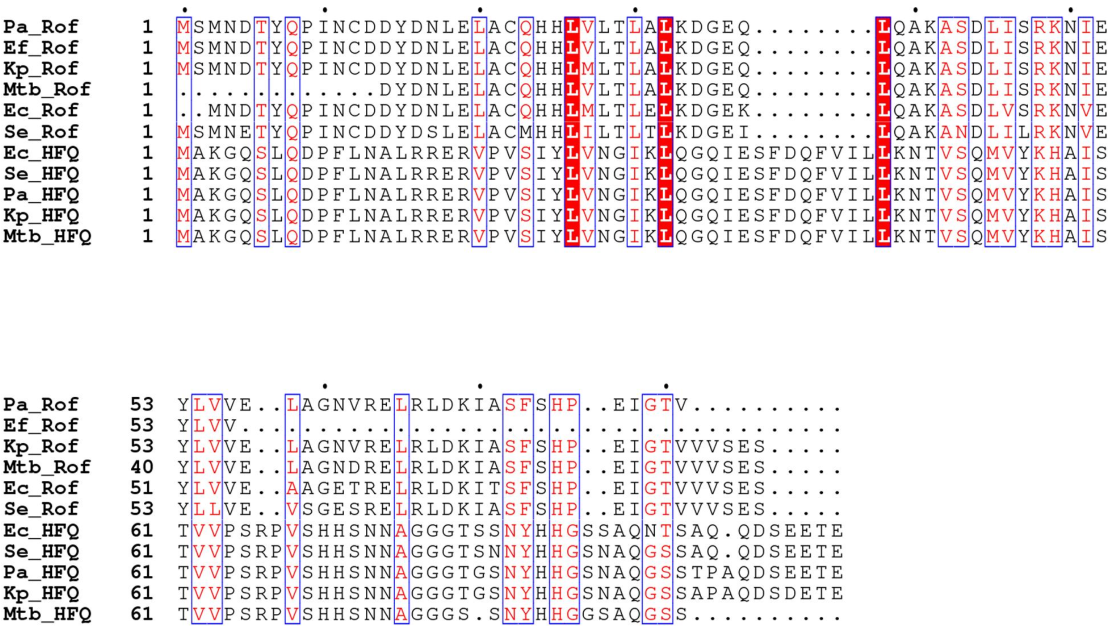

Supplementary Fig. 8. Sequence alignment of Rof and Hfq

Amino acid sequence alignment of Rof and Hfq from *Pa, Pseudomonas aeruginosa; Ef, Enterococcus faecalis; Kp, Klebsiella pneumoniae; Mtb, Mycobacterium tuberculosis; Se, Salmonella enterica; Ec, Escherichia_coli.*

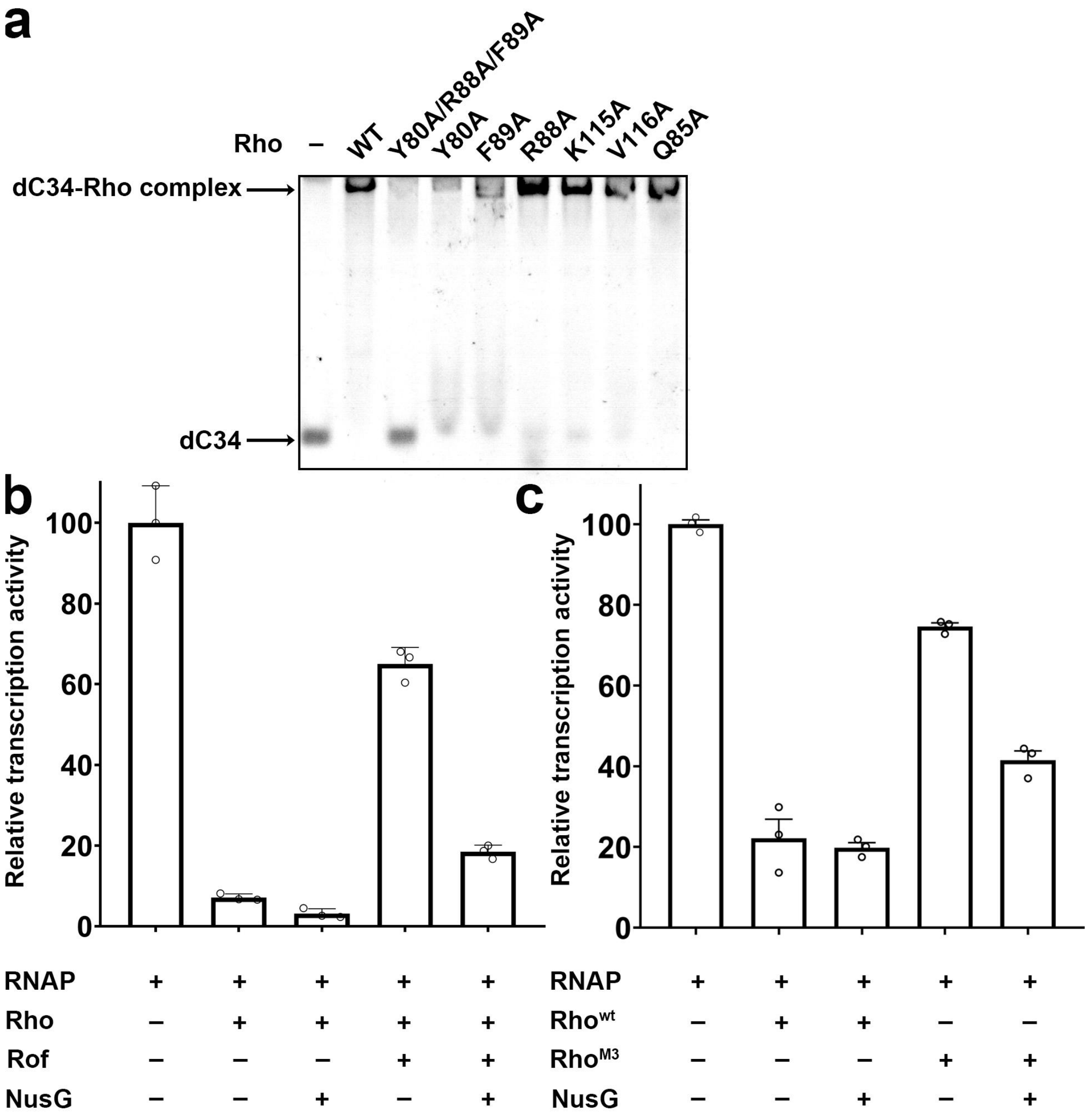

Supplementary Fig. 9. Relative *in vitro* transcription activity

(A) EMSA assay for dC34 binding with . Rho and 3μm dC34 were added into the reaction system. Bands of dC34, dC34-Rho complex are indicated by arrows on the left, respectively.

(B) Relative in *vitro* transcription activity showing NusG is functional in Rho-dependent termination. Data for *in vitro* transcription assays are means of three technical replicates. Error bars represent ± SEM of n = 3 experiments.

(C) Relative in *vitro* transcription activity with Rho and its mutation. Data for *in vitro* transcription assays are means of three technical replicates. Error bars represent ± SEM of n = 3 experiments.

Supplementary Table 1: Cryo-EM structure: E. coli Rho-Rof

	**Rho-Rof (EMDB-37342) (PDB 8W8D)**
**Data collection and processing**	
Magnification	105,000 x
Voltage (kV)	300
Electron exposure (e–/Å^2)	50
Defocus range (μm)	-0.8 to -2.0
Pixel size (Å)	1.19
Symmetry imposed	C1
Initial particle images (no.)	6,033
Final particle images (no.)	5,823
Map resolution (Å)	2.8
FSC threshold	0.143
Map resolution range (Å)	2.62-48
**Refinement**	
Initial model used (PDB code)	1PV4, 1SG5
Model resolution (Å)	3.1
FSC threshold	0.5
Model composition	
Non-hydrogen atoms	23034
Protein residues	2921
Ligands	N/A
B factors (Å)	
Protein	24.15
Ligands	N/A
R.m.s. deviations	
Bond lengths (Å)	0.007
Bond angles (°)	1.225
Validation	
MolProbity score	2.59
Clashscore	12.07
Poor rotamers (%)	1.25
Ramachandran plot	
Favored (%)	95.28
Allowed (%)	4.72
Disallowed (%)	0
