# Supplementary material for: A widely conserved protein Rof inhibits transcription termination factor Rho and promotes Salmonella virulence program
Source: Nat Commun. 2024 Apr 15;15:3187. doi: 10.1038/s41467-024-47438-7 (PMC11018607; doi:10.1038/s41467-024-47438-7)
Supplement: Supplementary file 3 — Reporting Summary [file 41467_2024_47438_MOESM3_ESM.pdf]

nature portfolio

Corresponding author(s): Chengyuan Wang

Last updated by author(s): Jan 29, 2024

Reporting Summary

Nature Portfolio wishes to improve the reproducibility of the work that we publish. This form provides structure for consistency and transparency in reporting. For further information on Nature Portfolio policies, see our Editorial Policies and the Editorial Policy Checklist.

Statistics

For all statistical analyses, confirm that the following items are present in the figure legend, table legend, main text, or Methods section.

n/a	Confirmed	
☐	☒	The exact sample size (*n*) for each experimental group/condition, given as a discrete number and unit of measurement
☐	☒	A statement on whether measurements were taken from distinct samples or whether the same sample was measured repeatedly
☐	☒	The statistical test(s) used AND whether they are one- or two-sided<br>*Only common tests should be described solely by name; describe more complex techniques in the Methods section.*
☐	☒	A description of all covariates tested
☐	☒	A description of any assumptions or corrections, such as tests of normality and adjustment for multiple comparisons
☐	☒	A full description of the statistical parameters including central tendency (e.g. means) or other basic estimates (e.g. regression coefficient) AND variation (e.g. standard deviation) or associated estimates of uncertainty (e.g. confidence intervals)
☐	☒	For null hypothesis testing, the test statistic (e.g. F, t, r) with confidence intervals, effect sizes, degrees of freedom and P value noted<br>*Give P values as exact values whenever suitable.*
☐	☒	For Bayesian analysis, information on the choice of priors and Markov chain Monte Carlo settings
☐	☒	For hierarchical and complex designs, identification of the appropriate level for tests and full reporting of outcomes
☐	☒	Estimates of effect sizes (e.g. Cohen's *d*, Pearson's *r*), indicating how they were calculated

Our web collection on statistics for biologists contains articles on many of the points above.

Software and code

Policy information about availability of computer code

Data collection	Serial-EM 4.0
Data analysis	Relion-3.0, WinCoot 0.8.9.2 EL, Phenix 1.20.1-4487, Chimera 1.14, Pymol 2.3

For manuscripts utilizing custom algorithms or software that are central to the research but not yet described in published literature, software must be made available to editors and reviewers. We strongly encourage code deposition in a community repository (e.g. GitHub). See the Nature Portfolio guidelines for submitting code & software for further information.

Data

Policy information about availability of data

All manuscripts must include a data availability statement. This statement should provide the following information, where applicable:

- Accession codes, unique identifiers, or web links for publicly available datasets
- A description of any restrictions on data availability
- For clinical datasets or third party data, please ensure that the statement adheres to our policy

Cryo-EM map have been deposited in the Electron Microscopy Database (EMDB accession code EMD-37342), and atomic coordinate have been deposited in the Protein Database (PDB accession code 8W8D).

Research involving human participants, their data, or biological material

Policy information about studies with human participants or human data. See also policy information about sex, gender (identity/presentation), and sexual orientation and race, ethnicity and racism.

Reporting on sex and gender	N/A
Reporting on race, ethnicity, or other socially relevant groupings	N/A
Population characteristics	N/A
Recruitment	N/A
Ethics oversight	N/A

Note that full information on the approval of the study protocol must also be provided in the manuscript.

Field-specific reporting

Please select the one below that is the best fit for your research. If you are not sure, read the appropriate sections before making your selection.

☒ Life sciences ☐ Behavioural & social sciences ☐ Ecological, evolutionary & environmental sciences

For a reference copy of the document with all sections, see nature.com/documents/nr-reporting-summary-flat.pdf

Life sciences study design

All studies must disclose on these points even when the disclosure is negative.

Sample size	Not relevant to methods used.
Data exclusions	None.
Replication	Experiments (Fig. 2, 3, 4, S5, S9) were performed three times with consistent results.<br>Experiments (Fig. 1) were performed twice with consistent results.
Randomization	For each assay, bacterial colonies have same genetic background and were randomly allocated to experimental groups.
Blinding	Not relevant to methods used.

Reporting for specific materials, systems and methods

We require information from authors about some types of materials, experimental systems and methods used in many studies. Here, indicate whether each material, system or method listed is relevant to your study. If you are not sure if a list item applies to your research, read the appropriate section before selecting a response.

Materials & experimental systems

n/a	Involved in the study	
☐	☒	Antibodies
☐	☒	Eukaryotic cell lines
☒	☐	Palaeontology and archaeology
☒	☐	Animals and other organisms
☒	☐	Clinical data
☒	☐	Dual use research of concern
☒	☐	Plants

Methods

n/a	Involved in the study	
☒	☐	ChIP-seq
☒	☐	Flow cytometry
☒	☐	MRI-based neuroimaging

Antibodies

Antibodies used	Polyclonal anti-GroEL antibody produced in rabbit, Sigma, #G6532, 1:10000;<br>Monoclonal anti-FLAG M2 antibody produced in mouse, Sigma, #F1804, 1:10000;<br>Polyclonal anti-SipC antibody produced in rabbit, gift from Joerg Vogel.

	Secondary HRP-linked goat anti-mouse IgG antibody, BBI, #D110087 1:10000;<br>Secondary HRP-linked goat anti-rabbit IgG antibody, BBI, #D110058, 1:10000;
Validation	We used commercial antibody reagents for Western-blot. Validation data are available on manufacturer's websites and data sheets.

Eukaryotic cell lines

Policy information about cell lines and Sex and Gender in Research

Cell line source(s)	HCT116 (#SCSP-644, Shanghai, China)
Authentication	None
Mycoplasma contamination	The cell line has been tested negative for mycoplasma contamination
Commonly misidentified lines<br>(See ICLAC register)	Not used

Plants

Seed stocks	*Report on the source of all seed stocks or other plant material used. If applicable, state the seed stock centre and catalogue number. If plant specimens were collected from the field, describe the collection location, date and sampling procedures.*
Novel plant genotypes	*Describe the methods by which all novel plant genotypes were produced. This includes those generated by transgenic approaches, gene editing, chemical/radiation-based mutagenesis and hybridization. For transgenic lines, describe the transformation method, the number of independent lines analyzed and the generation upon which experiments were performed. For gene-edited lines, describe the editor used, the endogenous sequence targeted for editing, the targeting guide RNA sequence (if applicable) and how the editor was applied.*
Authentication	*Describe any authentication procedures for each seed stock used or novel genotype generated. Describe any experiments used to assess the effect of a mutation and, where applicable, how potential secondary effects (e.g. second site T-DNA insertions, mosiacism, off-target gene editing) were examined.*
